# The Red Seaweed *Grateloupia turuturu* Prevents Epidermal Dysplasia in HPV16-Transgenic Mice

**DOI:** 10.3390/nu13124529

**Published:** 2021-12-17

**Authors:** José Almeida, Tiago Ferreira, Susana Santos, Maria J. Pires, Rui M. Gil da Costa, Rui Medeiros, Margarida M.S.M. Bastos, Maria J. Neuparth, Ana I. Faustino-Rocha, Helena Abreu, Rui Pereira, Mário Pacheco, Isabel Gaivão, Eduardo Rosa, Paula A. Oliveira

**Affiliations:** 1 Department of Veterinary Sciences, University of Trás-os-Montes and Alto Douro (UTAD), 5001-801 Vila Real, Portugal; josecfralmeida@gmail.com (J.A.); tiagoterras55@gmail.com (T.F.); suusanacoelhosantos@gmail.com (S.S.); mariomp@utad.pt (M.J.P.); anafaustino.faustino@sapo.pt (A.I.F.-R.); 2 Centre for the Research and Technology of Agro-Environmental and Biological Sciences (CITAB), Institute for Innovation, Capacity Building and Sustainability of Agri-Food Production (Inov4Agro), 5001-801 Vila Real, Portugal; rmcosta@fe.up.pt (R.M.G.d.C.); erosa@utad.pt (E.R.); 3 Maranhão Tumour and DNA Biobank (BTMA), Post-graduate Programme in Adult Health (PPGSAD), Federal University of Maranhão (UFMA), São Luís 65080-805, Brazil; 4 LEPABE—Laboratory for Process Engineering, Environment, Biotechnology and Energy, Faculty of Engineering, University of Porto, Rua Dr. Roberto Frias, 4200-465 Porto, Portugal; mbastos@fe.up.pt; 5 Molecular Oncology and Viral Pathology Group, Research Center of IPO Porto (CI-IPOP)/RISE@CI-IPOP (Health Research Network), Portuguese Oncology Institute of Porto (IPO Porto)/Porto Comprehensive Cancer Center (Porto. CCC), 4200-072 Porto, Portugal; ruimmms@gmail.com; 6 Faculty of Medicine, University of Porto (FMUP), 4200-450 Porto, Portugal; 7 CEBIMED, Faculty of Health Sciences, Fernando Pessoa University, 4200-150 Porto, Portugal; 8 LPCC Research Department, Portuguese League against Cancer (NRNorte), 4200-172 Porto, Portugal; 9 Research Center in Physical Activity, Health and Leisure (CIAFEL), Faculty of Sports, University of Porto, 4200-450 Porto, Portugal; mneuparth@hotmail.com; 10 Institute of Research and Advanced Training in Health Sciences and Technologies (IINFACTS), Advanced Polytechnic and University Cooperative (CESPU), 4585 Gandra, Portugal; 11 Department of Zootechnics, School of Sciences and Technology, 7000-671 Évora, Portugal; 12 ALGAplus, Lda., PCI-Creative Science Park, 3830-352 Ílhavo, Portugal; helena.abreu@algaplus.pt (H.A.); rui.pereira@algaplus.pt (R.P.); 13 A4F Algae for Future, Estrada do Paço do Lumiar, Campus do Lumiar, Edif. E, R/C, 1649-038 Lisboa, Portugal; 14 Portugal CESAM—Centre for Environmental and Marine Studies and Department of Biology, Santiago University Campus, University of Aveiro, 3810-193 Aveiro, Portugal; mpacheco@ua.pt; 15 Department of Genetic and Biotechnology, CECAV, UTAD, 5001-801 Vila Real, Portugal; igaivao@utad.pt; 16 Department of Agronomy, UTAD, 5001-801 Vila Real, Portugal

**Keywords:** HPV, in vivo, supplementation, chemoprevention, functional foods, natural products

## Abstract

The role of dietary profiles in promoting or reducing the risk of multiple types of cancer is increasingly clear, driving the search for balanced foods and nutraceuticals. The red seaweed *Grateloupia turuturu* has been used as human food showing a balanced nutritional profile. This study aims to test in vivo chemopreventive effects of *G. turuturu* against cutaneous pre-malignant lesions in transgenic mice for the human papillomavirus type 16 (HPV16). Forty-four female HPV^+/−^ or HPV^−/−^ mice received a standard diet or were supplemented with 10% *G. turuturu* for 22 consecutive days. Cutaneous lesions (ear and chest skin) were identified histologically. Complementarily, the weights and histology of internal organs as well as blood biochemical and DNA integrity parameters were also assessed. *G. turuturu* consistently reduced the incidence of epidermal dysplasia induced by HPV16 on both cutaneous sites. Moreover, biochemical, DNA integrity and histological analyses confirmed *G. turuturu* edibility as no signs of toxicity were found. Dietary supplementation with *G. turuturu* is an effective and safe chemopreventive strategy in this model.

## 1. Introduction

Seaweeds are very promising when it comes to their use as a source of primary and secondary bioactive metabolites [1,2,3]. They are rich in vitamins, minerals and fatty acids [4,5,6,7] and have long been used as a functional food, particularly in Asian countries, where they are associated with high average life expectancies [6]. The beneficial role of seaweeds against different neoplastic diseases, such as pancreatic, colorectal and breast cancer, has also found experimental support [8,9].

*Grateloupia turuturu* (phylum Rhodophyta), native from Japan, has been reported to typically contain only 2.6% lipids [10], which supports its use as food in the context of a healthy diet. A different study reported a higher lipid content varying between 3.3% and 4.1%, with polyunsaturated fatty acids accounting for 20.4% (winter harvest) to 31.1% (summer harvest) of the total [11]. Polysaccharides from *Grateloupia* spp. have shown interesting anti-neoplastic activities in vitro [12,13] and in vivo [12,14,15], suggesting that these seaweeds may be useful functional foods for cancer prevention, as part of a healthy lifestyle.

Cancer remains a major public health issue globally and 12% of registered cases are associated with biological carcinogens, such as viruses [16]. Among these viral carcinogenic agents, the human papillomavirus (HPV) occupies a prominent position, and is estimated to be responsible for 630,000 new cases of cancer per year [17]. Infection by HPV is responsible for cervical cancer in women, for other ano-genital cancers (e.g., anal cancer, penile cancer) and for a subset of head-and-neck cancers, leading to significant morbidity and mortality [17,18]. Through its oncoproteins, encoded by genes located in its early genomic region, high-risk HPVs are able to deregulate key cellular functions, leading to tumorigenesis, as recently reviewed [19]. There is an ongoing search for functional foods that may contribute to reducing cancer risk [20,21]. In this context, the main aim of this work was to evaluate the ability of *G. turuturu* to prevent the development of pre-malignant lesions in vivo, using a mouse model of cancers induced by HPV16 (K14HPV16 mice) [22]. Additionally, multiple toxicological parameters were assessed to evaluate this seaweed’s safety profile.

## 2. Materials and Methods

### 2.1. Animals

In this study, 44 female mice (*Mus musculus*) from an FVB/n background at 20 weeks of age were used. Transgenic (K14HPV16) mice carrying the whole HPV16 early region were generously donated by doctors Jeffrey Arbeit and Douglas Hanahan, from the University of California (USA), through the National Cancer Institute’s Mouse Repository. This model employs the human cytokeratin 14 gene promoter to direct the expression of all the early HPV16 genes to keratinized epithelia, producing proliferative lesions [22,23]. The animals were genotyped as previously described [24] and ascribed to transgenic or wild-type groups, as described under “Experimental design”. This study was carried out in accordance with Portuguese (Decree-Law nº133/2013) and European (Directive 2010/63/EU) legislation, after approval by the University of Trás-os-Montes and Alto Douro ethics committee (approval number 10/2013) and by the Portuguese Veterinary Directorate (approval number 0421/000/000/2014). During the study, the animals were provided water and food *ad libitum*.

### 2.2. G. turuturu Samples

The *G. turuturu* samples were harvested from Aguda beach (41°02′53.7″ N, 8°39′14.5″ W), Vila Nova de Gaia (NW coast of Portugal) in September 2015 and were identified by members of the seaweed aquaculture company ALGAplus (Ílhavo, Portugal).

The phytochemical profile of a *G. turuturu* batch harvested in the same geographic area as that of the present study (i.e., the NW coast of Portugal, at a distance of approximately 40 km) was previously studied using liquid chromatography and gas chromatography coupled with mass spectrometry and was characterized by an abundance of polar lipids (phospholipids, glycolipids, betaine lipids and phosphingolipids) with important antioxidant and anti-inflammatory properties [25]. In line with this, a *G. turuturu* batch from exactly the same site (i.e., Aguda beach, NW coast of Portugal) as that used in the present study, and harvested in the same month (i.e., September), showed favorable bioactivities and proved to be rich in bioactive compounds, in particular mycosporine-like amino acids such as shinorine, palythine, porphyra-334 and asterina-330 [26]. Therefore, it can be assumed that the current *G. turuturu* batch yields the same (or very similar) phytochemical profile as that described in the abovementioned studies [25,26].

### 2.3. Diet Preparation

After harvesting, seaweeds were washed with 5 µm filtered and UV-treated seawater and then dehydrated (24 h; 25 °C), freeze-dried (FTS Systems Dura-Dry MP, NY, USA; 1 week; 500 mTorr; −40 °C) and ground to a fine powder (0.5 mm in diameter). Thereafter, dried and grounded *G. turuturu* was mixed with a standard diet (Diet Standard 4RF21^®^, Ultragene, Italy), to obtain 2-mm-thick diet pellets containing 10% (*w*/*w*) of incorporated seaweed. This concentration was based on a previous *G. turuturu* dietary supplementation study [27]. The standard/control diet was processed similarly, except for the addition of *G. turuturu*. Finally, the pellets were dehydrated at 40 °C for 48 h and stored at 4 °C until consumed.

### 2.4. Experimental Design

Transgenic and wild-type animals were randomly divided into 4 experimental groups: Group I (HPV16^−/−^ with seaweed, *n* = 11) and group II (HPV16^+/−^ with seaweed, *n* = 11), which received the diet containing 10% *G. turuturu* supplementation; group III (HPV16^−/−^, *n* = 11) and group IV (HPV16^+/−^, *n* = 11), which were fed the standard diet, during 22 consecutive days. Weekly records of bodyweight as well as water and food consumption were kept. In addition, parameters related to animal welfare were evaluated during daily visits, including body condition, hair appearance, grooming behavior, the aspect of mucosae, the position of ears and whiskers, response to external stimuli, hydration status and the appearance of feces. At the end of the 22nd day, all animals were sacrificed by xylazine-ketamine overdose, followed by exsanguination by cardiac puncture, as recommended [28]. Blood samples were used for DNA integrity and biochemical analyses. Internal organs were weighed and fixed by immersion in 10% formaldehyde to study the possible toxic effects of *G. turuturu*. Skin samples from the chest and the left ear, two locations typically affected by HPV16 in this model [22], were collected, fixed and studied histologically to determine the evolution of tumor lesions and the chemopreventive effect of the seaweed.

### 2.5. Histological Analysis

After fixation, tissues were embedded in paraffin and 2-μm-thick sections were stained with hematoxylin and eosin (H&E). The skin samples were classified as normal, epidermal hyperplasia and dysplasia, based on the more advanced lesion present in each sample.

The normal epidermis showed 1 or 2 cell layers covered by a keratin layer. Both hyperplastic and dysplastic skin samples showed over 3 epidermal cell layers with a basaloid phenotype. Dysplasia occurred as foci within hyperplastic tissues and was further sub-classified as low-grade (occasional suprabasal mitotic figures, minimal dermal reactivity) or high-grade (numerous suprabasal mitotic figures, irregular and hyperchromatic nuclei, with close apposition of dermal blood capillaries) dysplasia [29].

### 2.6. Biochemical Analysis

Blood samples were centrifuged at 1400× *g*, for 15 min. Blood serum was used to quantify glucose, albumin, total proteins, alanine aminotransferase, aspartate aminotransferase and gamma-glutamyl transferase following spectrophotometric methods using an autoanalyzer (Prestige 24 i, Cormay PZ, Tokyo, Japan).

### 2.7. DNA Integrity Assessment

Two methods for DNA integrity assessment—the comet and the micronucleus assays—were performed on peripheral blood samples to determine whether *G. turuturu* supplementation in this model has a genoprotective effect. For each mouse, four glass slides were precoated with normal melting point agarose. Then, 10 µL of blood was mixed with 200 µL of phosphate buffered saline (PBS). After cell suspension preparation, 20 µL was mixed with 70 µL of 1% low melting point agarose, and two drops per slide were placed in each of our slides corresponding to each animal (11 animals per group). Then, the slides were immersed in a lysis solution (2.5 M NaCl, 0.1 M EDTA, 10 mM Tris, 1% Triton X-100, pH 10). For each animal, half of the slides were treated with formamidopyrimidine glycosilase (FPG) for 30 min. This enzyme is used to quantify oxidative damage in DNA, through the conversion of oxidized purines into DNA single-strand breaks, and was provided by Professor Andrew Collins (University of Oslo, Norway). Then, all the slides were treated with an alkaline solution (0.3 M NaOH and 1 mM EDTA, pH > 13). Using the same solution, the slides were electrophoresed at 25 V of voltage and 300 mA of current, for 30 min. After electrophoresis, the slides were neutralized in PBS, followed by distilled water and dehydrated in 70% ethanol and absolute ethanol. For the visualization and classification of comets, the slides were incubated with 1 µL/mL of 4′,6-diamidino-2-phenylindole (DAPI, Sigma-Aldrich Chemical Company, Spain). After this, the slides were observed under a fluorescence microscope Olympus BX41(Olympus America, Inc., Hauppauge, NY, USA), with a magnification of 400×. Comets were classified using Visual Comet Assay software into four categories that reflect DNA damage and vary between 0 (no tail) and 4 (almost all DNA present in the tail), with a count of 100 comets for each case [30]. The genetic damage index (GDI) was expressed in arbitrary units, according to the following formula:

GDI = (nucleoids class 0 × 0) + (nucleoids class 1 × 1) + (nucleoids class 2 × 2) + (nucleoids class 3 × 3) + (nucleoids class 4 × 4).

The micronucleus test was carried out on 2 blood smears per animal (11 animals per group). After drying at room temperature, the slides were fixed in methanol for 10 min, and stained with a 5% Giemsa solution for 30 min. The slides were observed in a bright field microscope (Nikon Eclipse E100), with a magnification of 1000×, and 1000 cells/slide were counted (2000 cells per animal), to determine the frequency of micronuclei.

### 2.8. Stastistical Analysis

The data were analyzed using Microsoft Excel and IBM SPSS Statistics software (Statistical Package for the Social Sciences, Chicago, IL, USA), version 25. The normality of data concerning organ relative weights, water and food consumption, blood biochemistry, DNA integrity and micronucleus frequency was confirmed using a Kolmogorov–Smirnov test. Then, an analysis of variance (ANOVA) was performed, followed by a Bonferroni test. Data from histology were compared through a chi-squared test. Differences were considered statistically significant when *p* < 0.05.

## 3. Results

During this study, all the animals survived until the end of the experimental period and did not reveal signs of distress according to any of the parameters monitored. There were no significant differences in terms of body weight between groups, nor significant differences regarding food and water consumption (data not shown).

### 3.1. Organ Relative Weights and Blood Biochemistry

The relative weight of internal organs (summarized in Table 1) as well as blood biochemical analyses (Table 2) showed no statistically significant differences (*p* > 0.05) between groups.

### 3.2. Histological Analysis

The results of the histological analysis of chest and ear skin samples are summarized in Table 3. Macroscopically, these lesions were crusting and thickened multifocal skin areas, associated with erythema. Epidermal hyperplasia and high-grade dysplasia were observed in the transgenic groups but not in wild-type animals. The modified diet containing *G. turuturu* drastically reduced the incidence of epidermal dysplasia at both cutaneous sites (Table 3), especially on the ear skin, which predominantly showed the less advanced hyperplastic phenotype (*p* = 0.024 for ear and *p* = 0.086 for chest).

### 3.3. DNA Integrity Assessment

**Figure 1:**
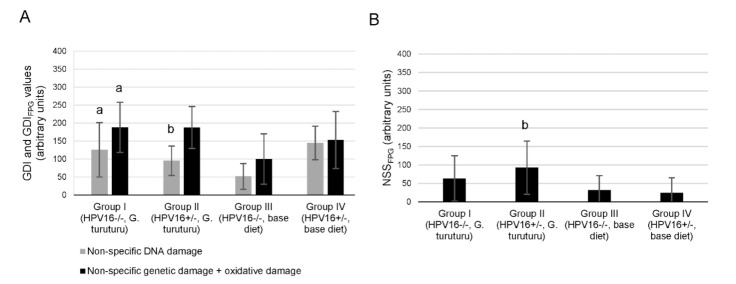
Analysis of DNA damage. (**A**) Mean ± standard deviation values of non-specific damage, expressed as genetic damage index (GDI, grey), and of non-specific plus oxidative damage, determined with formamidopyrimidine DNA glycosilase (FPG) and expressed as GDI_FPG_ (black), in white blood cells (*n* = 11/group). (**B**) Values of net FPG-sensitive sites (NSS_FPG_) from modified comet assay with FPG incubation to detect oxidized purine bases (obtained by the difference between GDI_FPG_ and GDI values). Statistically significant differences (*p* < 0.05): (a) Between group I and group III; (b) between group II and group IV.

**Figure 2:**
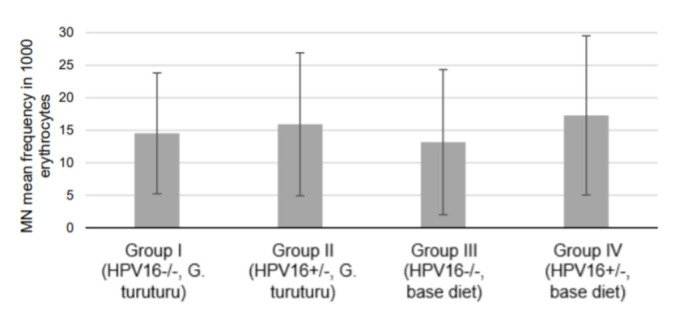
Frequency of micronuclei per 1000 erythrocytes (mean ± standard deviation; *n =* 11, two replicates per animal).

In the comet assay, a significant GDI decrease in transgenic mice supplemented with seaweed was observed compared with transgenic mice fed a standard diet (*p* = 0.025) (Figure 1A). Conversely, the wild-type group supplemented with seaweed (group I) showed a significant increase in the genetic damage (as GDI and GDI_FPG_) compared with the wild-type group fed the standard diet (group III) (*p* = 0.032 for and *p* = 0.042 GDI_FPG_) (Figure 1A). There was also a significant increase in the NSS_FPG_ level of the transgenic group supplemented with seaweed (group II) compared with the corresponding group fed with a standard diet (group IV; *p =* 0.038) (Figure 1B). In the micronucleus test, all experimental groups showed similar frequencies (Figure 2). 

## 4. Discussion

HPV is usually cleared quickly, but high-risk types are able to establish a persistent infection that may originate lesions that can progress to cancer. The burden of HPV infection and associated cancers remain high, especially in developing countries, where HPV screening and vaccination programs are often difficult to implement effectively [17]. Therefore, preventive strategies to reduce cancer risk by promoting healthy lifestyles, including the use of functional foods, are welcome. In the present study, we employed a well-characterized in vivo mouse model of lesions induced by HPV16 [22]. Transgenic mice carrying one, two or—as in this case—all the HPV16 oncogenes have been previously used by our research group and others to study the development of cervical [31], anal [32] and oral [33] cancers. These mice are also useful to test innovative chemopreventive strategies [34,35] and evaluate the toxic effects of harmful substances [36,37]. We observed a marked reduction in the progression of epidermal lesions in transgenic mice supplemented with *G. turuturu*, which showed only between one-third and one-sixth of the dysplastic lesions detected in matched non-supplemented mice.

In this animal model, epithelial lesions develop through intraepithelial stages before breaching the basement membrane and becoming invasive squamous cell carcinomas [22,31]. Those intraepithelial stages have been most often termed hyperplasia and dysplasia, although in a previous work specifically dealing with tongue base cancer, our group used the terms low-grade dysplasia and high-grade dysplasia [33]. For describing penile lesions, a number of designations taken from human pathology were adopted for comparative purposes [38]. In the present work, we adopted the widespread terms hyperplasia and dysplasia [22,29,31]. We attempted to further sub-classify dysplastic lesions into high-grade and low-grade dysplasia, but this proved unnecessary, as all dysplastic lesions fell into the high-grade category. Hyperplastic lesions merely show an expansion of the basal layers with maintained epithelial differentiation. In contrast, dysplastic lesions in this model represent a pivotal step for carcinogenesis with the acquisition of critical traits of malignancy, such as the ability to promote angiogenesis, demonstrated by the accumulation of capillaries in the superficial dermis in close contact with dysplastic foci [29]. Thus, the replacement of dysplasia by the less aggressive hyperplastic lesions in transgenic mice fed *G. turuturu* supports the hypothesis that dietary supplementation with this seaweed is able to attenuate the histopathological effects of the HPV16 oncogenes [20,39]. Of note, *G. turuturu* did not induce cutaneous lesions in wild-type mice, which is consistent with a favorable toxicological profile. In our previous study with another red seaweed and this animal model, we observed that *Porphyra umbilicalis* can completely block the development of dysplastic epidermal lesions in the chest skin [39]. The mechanisms whereby *G. turuturu* was able to block the development of HPV16-induced lesions remain unclear and require additional studies to clarify this point. In this direction, several marine algae displayed antioxidative and anti-inflammatory properties [40]. In fact, natural and synthetic compounds with anti-inflammatory properties have been repeatedly shown to prevent tumor development in this animal model [34,35,41,42,43]. The results also indicate that supplementation with 10% of *G. turuturu* for 22 days did not influence the survival, well-being or physiological parameters of mice, including water and food intake and body weight. In this context, serum biochemical and hepatic histological analyses did not suggest any toxicity or changes to liver function. This is an important point since this animal model is characterized by developing chronic systemic inflammation with hepatitis [35]. Indeed, *G. turuturu* helped in normalizing the relative weight of the liver, suggesting it exerted a protective effect.

Two genotoxicity tests were performed, which are recognized as two of the most consistent, sensitive and higher-statistical-power diagnostic tools in this field [44]. In the comet assay, the damage presented in the DNA is evaluated in terms of genomic integrity, after undergoing electrophoresis. This is carried out with alkaline pH, to facilitate and make the detection of DNA single and double-strand breaks more sensitive [45]. Hence, comet assay data evidenced the genoprotective potential of *G. turuturu* in transgenic animals, translated in the ability to reduce GDI levels to one-third compared to the non-supplemented group. In line with these results, other studies with marine macroalgae supplementation (including red seaweeds) in *Drosophila melanogaster* and gilthead seabream (*Sparus aurata*) reported genoprotective effects against endogenous and exogenous challenges [46,47].

The results concerning wild-type animals should be interpreted cautiously, since a hasty and superficial interpretation could lead to the definitive assumption that seaweed reduces DNA integrity. However, it must be considered that comet assay data reflect subtle variations resulting from a delicate balance between pro-genotoxic and anti-genotoxic pressures, as well as from the combined action of various phytochemicals present in the macroalga, which may work synergistically, antagonistically or additively. It has been demonstrated that substances (e.g., foods, beverages, natural medicinal products, extracts or isolated compounds) described as beneficial apparently showed a slight initial genotoxic action [48,49]. Moreover, this phenomenon was previously reported in a dietary assay with *D. melanogaster* following supplementation with the green macroalga *Ulva rigida* that, in parallel, unequivocally demonstrated a genoprotective action [49]. The rationale for this particular response profile (the so-called antioxidant hypothesis) states that a pro-oxidant potential can be beneficial, since mild oxidative stress may trigger cell antioxidant defenses and xenobiotic metabolizing enzymes, and, ultimately, contribute to preventing the development of certain diseases, such as cancer [50,51]. This mechanistic pattern is also plausible to explain the DNA strand break increase currently observed in the transgenic supplemented group resulting specifically from FPG (NSS_FPG_).

It is well established by publications relating to human trials that putative cancer-protective agents (e.g., polyphenols), in the presence of known DNA-damaging agents, show protection, as well as that low concentrations tend to improve DNA integrity, while high concentrations can themselves induce DNA damage [49]. Interestingly, this ambivalence of effects was called the “mixed blessing” by Azqueta and Collins [49] and represents a challenging topic in the field of functional foods.

Keeping in mind that DNA damage is the initiating event of carcinogenesis, the decrease in DNA damage currently observed in the transgenic supplemented group, in concomitance with the reduction in the progression of epidermal dysplastic lesions, highlights the potential of *G. turuturu* to reduce cancer risk in organisms under preneoplastic conditions. Keeping in mind that genoprotection involves a complex network of processes and must be regarded as a multiphasic action [52], the current comet assay data concerning wild-type animals point out the need to carry out experiments over a range of *G. turuturu* concentrations, which will be particularly relevant to allow result extrapolation to humans and the unequivocal recommendation of a regular consumption by healthy people.

Nevertheless, the combined analysis of all the genetic integrity endpoints assessed, particularly the absence of significant alterations on micronuclei frequency (an indicator of definitive and irreparable DNA damage), provides strong evidence that *G. turuturu* supplementation does not raise food safety issues.

## 5. Conclusions

Dietary supplementation with *G. turuturu* was highly effective towards the mitigation of the severity of histopathological alterations, namely preventing the development of HPV-16 induced dysplastic skin lesions, but the underlying mechanisms remain to be understood and additional studies are warranted to clarify this point. This seaweed showed a favorable toxicological profile, also revealing potential to protect DNA integrity in transgenic mice.

## Figures and Tables

**Table 1:** Relative weight (g/g) of collected organs in experimental groups (mean ± standard error).

	*Grateloupia turuturu*		Standard Diet	
	Group I (HPV16^−/−^)	Group II (HPV16^+/−^)	Group III (HPV16^−/−^)	Group IV (HPV16^+/−^)
Liver	0.0609 ± 0.0013	0.0688 ± 0.0049	0.0574 ± 0.0012	0.0717 ± 0.0185
Right Kidney	0.0063 ± 0.0001	0.0069 ± 0.0002	0.0057 ± 0.0002	0.0069 ± 0.0002
Left Kidney	0.0067 ± 0.0001	0.0070 ± 0.0003	0.0062 ± 0.0002	0.0068 ± 0.0002
Thymus	0.0012 ± 0.0001	0.0012 ± 0.0001	0.0012 ± 0.0002	0.0014 ± 0.0001
Heart	0.0048 ± 0.0001	0.0049 ± 0.0002	0.0048 ± 0.0002	0.0051 ± 0.0002
Lungs	0.0065 ± 0.0003	0.0060 ± 0.0004	0.0063 ± 0.0003	0.0071 ± 0.0002
Bladder	0.0086 ± 0.0070	0.0009 ± 0.0001	0.0003 ± 0.0002	0.0008 ± 0.0001
Spleen	0.0052 ± 0.0003	0.0066 ± 0.0005	0.0047 ± 0.0002	0.0083 ± 0.0010

**Table 2:** Serum biochemical parameters analyzed (mean ± standard error).

	*Grateloupia turuturu*		Standard Diet	
	Group I (HPV16 ^−/−^)	Group II (HPV16 ^+/−^)	Group III (HPV16 ^−/−^)	Group IV (HPV16 ^+/−^)
Albumin (g/L)	31.48 ± 0.69	30.64 ± 0.75	29.78 ± 1.71	30.37 ± 0.96
Total proteins (g/L)	50.11 ± 2.04	53.65 ± 1.63	51.34 ± 4.07	49.62 ± 1.12
Glucose (mg/dL)	234.96 ± 17.92	185.75 ± 9.06	195.70 ± 15.99	198.07 ± 13.36
Alanine aminotransferase (U/L)	30.77 ± 5.32	36.59 ± 4.27	37.28 ± 4.70	41.87 ± 3.54
Aspartate aminotransferase (U/L)	64.96 ± 8.31	67.03 ± 8.45	44.74 ± 3.76	51.82 ± 3.70
Gamma-glutamyl transferase (U/L)	33.39 ± 3.70	36.75 ± 4.00	48.61 ± 6.11	60.78 ± 8.35

**Table 3:** Incidence of histological lesions in chest and ear skin in the four experimental groups.

		*G. turuturu* Supplementation		Standard Diet	
		Group I (HPV16^−/−^)	Group II (HPV16^+/−^)	Group III (HPV16^−/−^)	Group IV (HPV16^+/−^)
Chest skin affected mice/n (%)	Normal	11/11 (100.0%)	0/10 (0.0%)	11/11 (100.0%)	0/11 (0.0%)
	Epidermal Hyperplasia	0/11 (0.0%)	8/10 (80.0%)	0/11 (0.0%)	4/11 (36.4%)
	Epidermal Dysplasia	0/11 (0.0%)	2/10 (20.0%)	0/11 (0.0%)	7/11 (63.6%)
Ear affected mice/n (%)	Normal	11/11 (100.0%)	0/10 (0.0%)	11/11 (100.0%)	0/11 (0.0%)
	Epidermal Hyperplasia	0/11 (0.0%)	9/10 (90.0%) ^a^	0/11 (0.0%)	4/11 (36.4%)
	Epidermal Dysplasia	0/11 (0.0%)	1/10 (10.0%) ^a^	0/11 (0.0%)	7/11 (63.6%)

^a^ Statistically significant difference between group II and group IV (*p* < 0.05).

## Data Availability

The data presented in this study are available on request from the corresponding author.
